# Supplementary material for: Smoking does not accelerate leucocyte telomere attrition: a meta-analysis of 18 longitudinal cohorts
Source: R Soc Open Sci. 2019 Jun 5;6(6):190420. doi: 10.1098/rsos.190420 (PMC6599800; doi:10.1098/rsos.190420)
Supplement: Supplementary tables and figures [file rsos190420supp1.pdf]

Supplementary information for *Royal Society Open Science* article:

Smoking does not accelerate leukocyte telomere attrition: a meta-analysis of 18 longitudinal cohorts

Melissa Bateson[1*], Abraham Aviv[2], Laila Bendix[3], Athanase Benetos[4], Yoav Ben-Shlomo[5], Stig E. Bojesen[6], Cyrus Cooper[7], Rachel Cooper[8], Ian J. Deary[9], Sara Hägg[10], Sarah E. Harris[9,11], Jeremy D. Kark[12], Florian Kronenberg[13], Diana Kuh[8], Carlos Labat[14], Carmen M. Martin-Ruiz[1], Craig Meyer[15], Børge G. Nordestgaard[6], Brenda W. J. H. Penninx[16], Gillian V. Pepper[1], Dóra Révész[17], M. Abdullah Said[18], John M. Starr[9,19,†], Holly Syddall[7], William Murray Thomson[20], Pim van der Harst[18], Mary Whooley[15], Thomas von Zglinicki[21,22], Peter Willeit[23,24], Yiqiang Zhan[10] and Daniel Nettle[1]

1 Centre for Behaviour and Evolution and Institute of Neuroscience, Newcastle University, Newcastle upon Tyne, NE2 4HH, United Kingdom.
2 Center of Human Development and Aging, New Jersey Medical School, Rutgers University, Newark, New Jersey, 07103, USA.
3 Pain Center South, Department of Anesthesiology and Intensive Care Medicine, University Hospital Odense, Odense, Denmark.
4 Department of Geriatric Medicine, CHRU de Nancy, Université de Lorraine, Nancy, France.
5 School of Social and Community Medicine, University of Bristol, Canynge Hall, Bristol, United Kingdom.
6 Department of Clinical Biochemistry, Herlev and Gentofte Hospital, Copenhagen University Hospital, Copenhagen University, Copenhagen, DK-2730 Herlev, Denmark.
7 MRC Lifecourse Epidemiology Unit, University of Southampton, Southampton General Hospital, Southampton, SO16 6YD, United Kingdom.
8 MRC Unit for Lifelong Health and Ageing at UCL, University College London, 33 Bedford Place, London, WC1B 5JU, United Kingdom.
9 Centre for Cognitive Ageing and Cognitive Epidemiology, Department of Psychology, University of Edinburgh, Edinburgh, EH8 9JZ, United Kingdom.
10 Department of Medical Epidemiology and Biostatistics, Karolinska Institutet, 171 77 Stockholm, Sweden.
11 Medical Genetics Section, University of Edinburgh Centre for Genomic and Experimental Medicine and MRC Institute of Genetics and Molecular Medicine, Edinburgh, EH4 2XU, United Kingdom.
12 Hebrew University–Hadassah School of Public Health and Community Medicine, Ein Kerem, Jerusalem, Israel.
13 Division of Genetic Epidemiology, Department of Medical Genetics, Molecular and Clinical Pharmacology, Innsbruck Medical University, Innsbruck, 6020, Austria.
14 INSERM U1116, Université de Lorraine; Nancy, France.
15 Department of Medicine, University of California, San Francisco, CA, 94121, USA.
16 Department of Psychiatry, VU University Medical Center, Oldenaller 1, 1081 HJ Amsterdam, The Netherlands.
17 Department of Epidemiology, GROW School for Oncology and Developmental Biology, Maastricht University, P.O. Box 616, 6200 MD, Maastricht, The Netherlands.
18 University of Groningen, University Medical Center Groningen, Department of Cardiology, Groningen, 9700RB, The Netherlands.
19 Alzheimer Scotland Dementia Research Centre, University of Edinburgh, Edinburgh, EH8 9JZ, United Kingdom.
20 Sir John Walsh Research Institute, Faculty of Dentistry, University of Otago, Dunedin, 9054, New Zealand.
21 Institute for Cell and Molecular Biosciences, Newcastle University, Newcastle upon Tyne, United Kingdom.
22 Near East University, Arts and Sciences Faculty, Molecular Biology and Genetics, Nicosia, North Cyprus, Mersin 10, Turkey.

23 Department of Neurology, Medical University of Innsbruck, Innsbruck, 6020, Austria
24 Department of Public Health and Primary Care, University of Cambridge, Cambridge, United Kingdom.

† John Starr died whilst this paper was under review and the final version was prepared posthumously.

*Corresponding author:

Professor Melissa Bateson

Email: melissa.bateson@ncl.ac.uk

Phone: +44 191 2085056

ORCID ID: 0000-0002-0861-0191

Contents

Page 3: **Table S1. Summary of LTL data used in the meta-analysis.**

Page 4: **Table S2.** Results from the leave-one-out sensitivity analysis of model 6.

Page 5: **Fig. S1. PRISMA diagram detailing the source of the data for the meta-analysis.** Nine of the cohorts included in the final meta-analysis were identified via an initial systematic literature search and the other nine were identified via a process of snowballing.

Page 6: **Fig. S2. LTL decreases with increasing age.** (a) Forest plot showing that LTL is significantly shorter at follow-up compared to baseline. For key see Fig. 2. (b) Scatterplot showing that longer follow-up intervals are associated with a greater decline in LTL between baseline and follow-up. The black line shows the estimate from a random-effects meta-regression obtained by adding mean follow-up interval as a moderator to model 1; this estimate is based on all 18 cohorts in the plot. For key see Fig. 3b. The additional blue line shows the estimate from the same meta-regression (±95% CI) based on the subset of cohorts measured using TRF.

Supplementary Information: Table S1. Summary of LTL data used in the meta-analysis.

**Cohort**[1]	**LTL measurement**		**Baseline TL**				**Follow-up TL**				**Telomere attrition (/year)**				**Pearson correlation between baseline and follow-up LTL**	
	**Method**[2]	**Units**[3]	**Smokers**		**Non-smokers**		**Smokers**		**Non-smokers**		**Smokers**		**Non-smokers**		**r**	**p-value**
			**Mean**	**sd**	**Mean**	**sd**	**Mean**	**sd**	**Mean**	**sd**	**Mean**	**sd**	**Mean**	**sd**		
ADE	TRF	bp	6290.00	721.25	6378.57	465.85	6155.00	855.60	6128.57	490.83	16.44	15.56	30.02	24.20	0.92	<0.0001*
BHS	TRF	bp	7392.00	777.00	7481.00	777.00	7150.00	772.00	7270.00	772.00	42.00	46.00	40.00	46.00	0.95	<0.0001*
BRUNECK	qPCR	T/S	1.64	0.83	1.68	0.84	1.15	0.61	1.18	0.55	0.05	0.06	0.05	0.06	0.64	<0.0001*
CCHS	qPCR	bp	4318.00	1047.00	4434.00	1062.00	4042.00	1010.00	4116.00	1031.00	28.00	120.00	33.00	122.00	0.39	<0.0001*
CCS	qPCR	bp	4302.39	1591.62	4506.89	1867.65	3438.83	1472.86	3080.53	938.18	112.76	288.71	183.06	261.03	0.03	0.5990
DMHDS	qPCR	T/S	1.17	0.37	1.20	0.41	1.02	0.31	1.05	0.31	0.01	0.02	0.01	0.03	0.66	<0.0001*
ERA	TRF	bp	5860.00	0.00	6428.84	620.03	5710.00	0.00	6198.26	601.17	16.04	0.00	24.34	15.52	0.97	<0.0001*
ESTHER	qPCR	bp	5940.00	384.49	6020.00	315.18	5190.00	522.04	5810.00	616.72	1.30	84.35	14.50	99.80	NA	NA
HAS	qPCR	bp	5490.26	1464.64	5417.45	1443.86	3637.15	1539.88	4048.20	1446.62	204.74	265.92	149.04	236.01	-0.16	0.0919
HSS	qPCR	bp	5546.32	543.21	5483.28	527.85	5325.56	406.29	5277.76	344.28	46.12	119.89	42.05	102.77	0.40	<0.0001*
JLRCS	TRF	bp	7253.00	650.00	7361.00	673.00	6907.00	590.00	7053.00	644.00	26.50	14.00	23.50	13.60	0.96	<0.0001*
LBC1921	qPCR	bp	3912.37	301.05	4096.44	457.96	3290.00	770.88	3542.61	791.46	49.64	33.08	64.34	138.97	0.35	0.0017*
LBC1936	qPCR	bp	4073.34	579.07	4207.34	571.33	3680.81	648.08	3805.03	708.36	86.94	119.95	66.38	112.14	0.54	<0.0001*
MONICA	qPCR	T/S	0.75	0.16	0.74	0.16	0.65	0.19	0.67	0.20	0.01	0.02	0.01	0.02	0.37	<0.0001*
NESDA	qPCR	bp	5407.29	623.56	5511.60	614.67	5370.51	403.03	5440.66	446.86	6.13	96.55	11.82	95.26	0.45	<0.0001*
NSHD	qPCR	bp	5610.81	2017.47	5792.41	1827.71	4241.11	1236.59	4297.16	1345.08	148.61	235.88	160.14	234.61	0.12	0.0102*
PREVEND	qPCR	T/S	1.05	0.32	1.09	0.32	1.00	0.36	1.07	0.35	0.01	0.07	0.00	0.07	0.13	<0.0001*
SATSA	qPCR	T/S	0.73	0.19	0.73	0.22	0.72	0.15	0.74	0.17	0.00	0.03	0.00	0.03	0.39	<0.0001*

[1]Full details of each cohort are provided in Table 1; [2]Measurement methods for LTL: TRF = terminal restriction fragment and qPCR = quantitative polymerase chain reaction; [3]Units of LTL measurement: bp = base pairs and T/S = T/S ratios.

Supplementary Information: Table S2. Results from the leave-one-out sensitivity analysis of model 6.

Cohort omitted	Summary SMD		Heterogeneity statistics				
	**Estimate* [95% CI]**	**p-value**	**Q_{16}**	**p-value**	**τ^2**	**I^2 (%)**	**H^2**
ADE	-0.02 [-0.07, 0.04]	0.5116	24.06	0.0881	0.00	37.93	1.61
BHS	-0.02 [-0.08, 0.05]	0.6091	24.56	0.078	0.01	41.03	1.70
BRUNECK	-0.02 [-0.08, 0.04]	0.5257	24.64	0.0764	0.00	40.44	1.68
CCHS	-0.03 [-0.09, 0.03]	0.3793	21.65	0.155	0.00	32.98	1.49
CCS	-0.03 [-0.08, 0.03]	0.2978	19.97	0.2215	0.00	30.71	1.44
DMHDS	-0.02 [-0.08, 0.04]	0.5217	24.61	0.077	0.01	40.93	1.69
ERA	-0.02 [-0.07, 0.04]	0.5214	24.39	0.0814	0.00	37.93	1.61
ESTHER	-0.03 [-0.08, 0.03]	0.3552	22.49	0.1282	0.00	34.74	1.53
HAS	-0.02 [-0.07, 0.04]	0.5973	23.93	0.091	0.00	38.05	1.61
HSS	-0.02 [-0.08, 0.04]	0.5721	24.64	0.0764	0.00	40.12	1.67
JLRCS	-0.01 [-0.06, 0.05]	0.8152	20.47	0.1996	0.00	29.70	1.42
LBC1921	-0.02 [-0.07, 0.04]	0.5256	24.64	0.0765	0.00	38.03	1.61
LBC1936	-0.01 [-0.07, 0.05]	0.6828	23.16	0.1096	0.00	36.99	1.59
MONICA	0.00 [-0.05, 0.05]	0.9947	18.39	0.3017	0.00	13.68	1.16
NESDA	-0.03 [-0.09, 0.03]	0.3918	23.27	0.1068	0.00	36.71	1.58
NSHD	-0.02 [-0.08, 0.04]	0.4743	24.33	0.0825	0.00	39.61	1.66
PREVEND	-0.01 [-0.08, 0.05]	0.6692	23.98	0.09	0.01	38.68	1.63
SATSA	-0.02 [-0.07, 0.04]	0.6088	24.25	0.0841	0.00	38.80	1.63

*Negative parameter estimates for the summary standardized mean difference (SMD) correspond to faster attrition in smokers. The cohort with the largest influence is shaded.

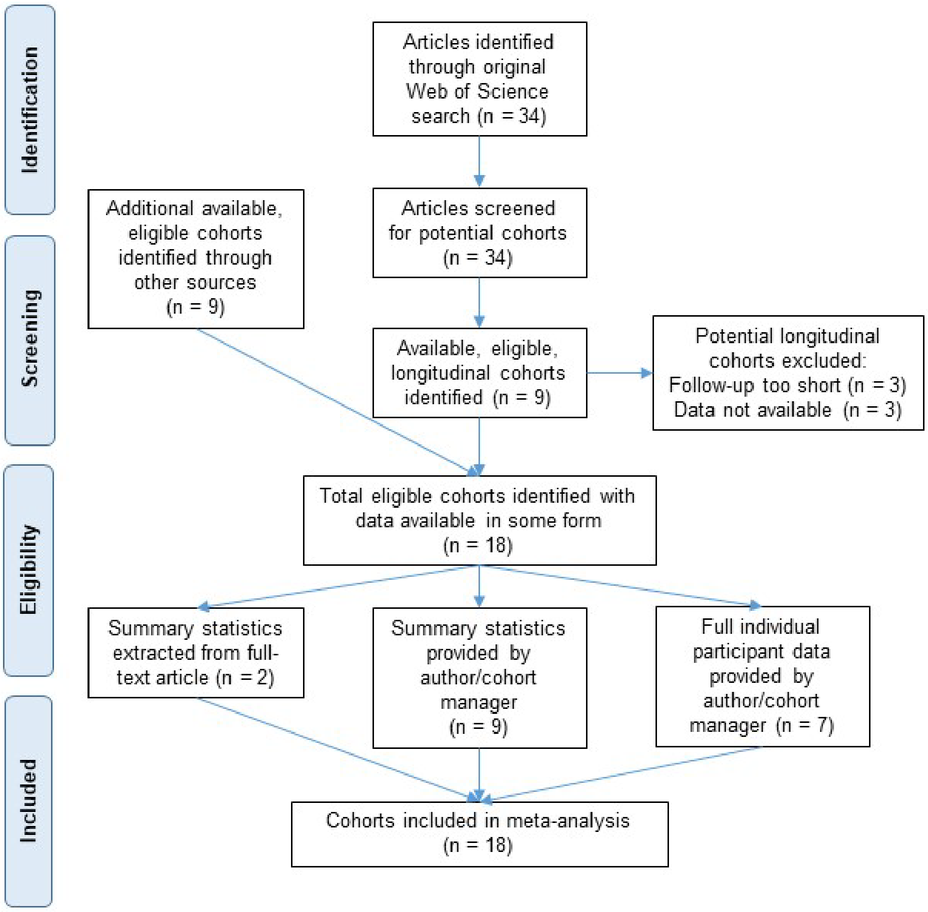

Supplementary Information: Fig. S1. PRISMA diagram detailing the source of the data for the meta-analysis. Nine of the cohorts included in the final meta-analysis were identified via an initial systematic literature search and the other nine were identified via a process of snowballing.

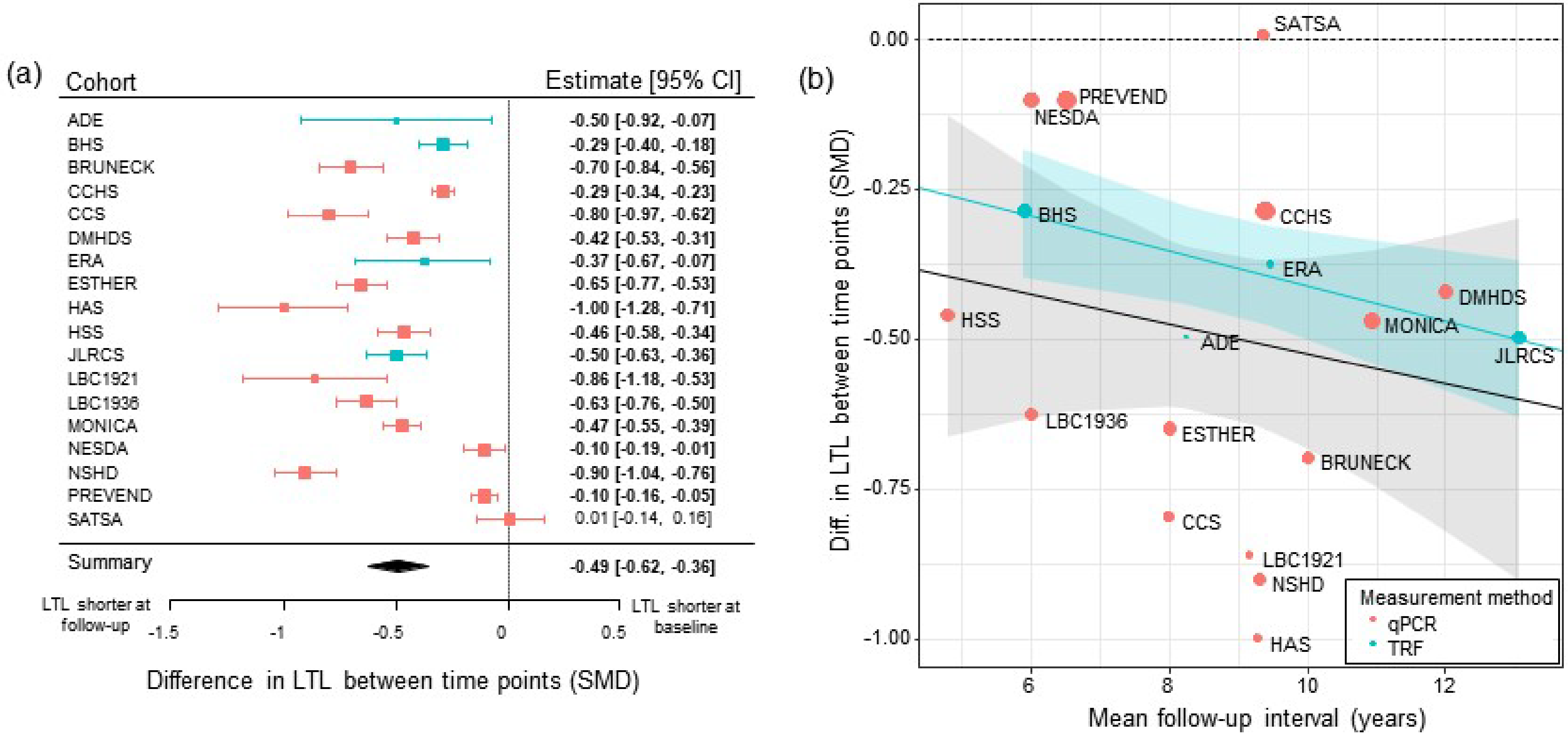

Supplementary Information: Fig. S2. LTL decreases with increasing age. (a) Forest plot showing that LTL is significantly shorter at follow-up compared to baseline. For key see Fig. 2. (b) Scatterplot showing that longer follow-up intervals are associated with a greater decline in LTL between baseline and follow-up. The black line shows the estimate from a random-effects meta-regression obtained by adding mean follow-up interval as a moderator to model 1; this estimate is based on all 18 cohorts in the plot. For key see Fig. 3b. The additional blue line shows the estimate from the same meta-regression (±95% CI) based on the subset of cohorts measured using TRF.
